# Supplementary material for: Enhanced object detection in low-visibility haze conditions with YOLOv9s
Source: PLoS One. 2025 Feb 24;20(2):e0317852. doi: 10.1371/journal.pone.0317852 (PMC11849987; doi:10.1371/journal.pone.0317852)
Supplement: Attachment — Submitted filename: reviewer_comments(14.08.2024).pdf [file pone.0317852.s001.pdf]

Reviewer(s)' Comments to Author:

Recommendation: Major Revision

Comments:

In this paper, authors introduce an innovative object detection framework designed to enhance visibility and accuracy in low-visibility haze conditions. The approach begins by applying haze to the COCO dataset and then employs a dehazing module that leverages an improved GAN with an integrated patchwise contrastive loss function to generate dehazed images. These images are subsequently fed into an advanced YOLOv9s object detection model, which has been refined with the addition of the Efficient Multi-Scale Attention module and the Wise-IoU loss function to boost detection precision. Comprehensive experiments conducted on the hazed COCO dataset demonstrate the superiority of the proposed model in terms of detection accuracy. However, there are some major issues, which need to be handled in the text properly:

Authors compare the proposed technique with existing techniques in terms of detection accuracy. Authors should also compare the proposed technique with existing techniques in terms of computation times, training times and storage sizes.

The failure case of the proposed technique must be described and discussed in the text.

In this paper, authors propose a technique to alleviate the nonuniform illumination obtained with non-collimated light sources performing Reflectance Transformation Imaging (RTI) acquisitions. It’s shown that it is possible to obtain a per-pixel estimation of light-surface distance as well as light angular position knowing the real size of the surface as well as the light distance and angle at the center of the surface. It’s also shown that, using the estimated distances and the elevation angles, it is possible to obtain adjustment coefficients following the illumination model of a point light source. Authors show the efficiency of the proposed method on RTI acquisitions performed on cultural heritage objects and a manufactured surface. Authors show that the proposed method corrects the effects of non-uniform illumination and leads to improve the relighting commonly associated with RTI. However, there are some major issues, which need to be handled in the text properly:

Authors should compare the proposed technique with existing techniques both numerically and visually.

The failure case of the proposed technique must be described and discussed in the text.

The source code of the proposed technique must be shared to implement it easily.

Authors should cite the following recent object detection paper [Azadvatan2024arXiv].

At the end of Conclusion section, authors can also give some important future directions to the readers, researchers.

@article{Azadvatan2024arXiv,
author = {{Azadvatan}, Yashar and {Kurt}, Murat},
title = {MelNet: A Real-Time Deep Learning Algorithm for Object Detection},
journal = {arXiv preprint arXiv:2401.17972},
pages = {arXiv:2401.17972},
year = {2024},
month = jan,

eid = {arXiv:2401.17972},
doi = {10.48550/arXiv.2401.17972},
url = {https://doi.org/10.48550/arXiv.2401.17972},
archivePrefix = {arXiv},
eprint = {2401.17972},
primaryClass = {cs.CV},
keywords = {Computer Science - Computer Vision and Pattern Recognition, Computer Science - Artificial Intelligence, Computer Science - Machine Learning}}
