# Supplementary material for: Enhanced object detection in low-visibility haze conditions with YOLOv9s
Source: PLoS One. 2025 Feb 24;20(2):e0317852. doi: 10.1371/journal.pone.0317852 (PMC11849987; doi:10.1371/journal.pone.0317852)
Supplement: Attachment — Submitted filename: reviewer_comments(02.12.2024).pdf [file pone.0317852.s003.pdf]

Reviewer(s)' Comments to Author:

Recommendation: Minor Revision

Comments:

In this paper, authors introduce an innovative object detection framework designed to enhance visibility and accuracy in low-visibility haze conditions. The approach begins by applying haze to the COCO dataset and then employs a dehazing module that leverages an improved GAN with an integrated patchwise contrastive loss function to generate dehazed images. These images are subsequently fed into an advanced YOLOv9s object detection model, which has been refined with the addition of the Efficient Multi-Scale Attention module and the Wise-IoU loss function to boost detection precision. Comprehensive experiments conducted on the hazed COCO dataset demonstrate the superiority of the proposed model in terms of detection accuracy. In the revised paper, it seems that authors handled most of the reviewer comments into account. However, there are some minor issues, which need to be handled in the text properly:

Authors compare the proposed technique with existing techniques numerically. Authors should also compare the proposed technique with existing techniques visually.
