# Supplementary material for: Enhanced object detection in low-visibility haze conditions with YOLOv9s
Source: PLoS One. 2025 Feb 24;20(2):e0317852. doi: 10.1371/journal.pone.0317852 (PMC11849987; doi:10.1371/journal.pone.0317852)
Supplement: Attachment — Submitted filename: response to reviewers.docx [file pone.0317852.s002.docx]

**Original Manuscript ID:** PONE-D-24-31889

**Original Article Title: “**Enhanced Object Detection in Low-Visibility Haze Conditions with YOLOv9s”

**To:** PLOS ONE Editor

**Re:** Response to reviewers

Dear Editor,

Thank you for allowing a resubmission of our manuscript, with an opportunity to address the reviewers’ comments.

We are uploading (a) our point-by-point response to the comments (below) (Response to Reviewers) and (b) an updated manuscript with blue highlighting indicating changes (Revised Manuscript with Track Changes) and (c) an updated manuscript without blue highlighting indicating changes (Manuscript).

Best regards,

Bin Zhou et al.

Journal Requirements:

**Q1.** We note the following restriction on images sourced from the COCO Consortium:

- "The COCO Consortium does not own the copyright of the images. Use of the images must abide by the Flickr Terms of Use. The users of the images accept full responsibility for the use of the dataset, including but not limited to the use of any copies of copyrighted images that they may create from the dataset." (https://cocodataset.org/#termsofuse)

As such, we cannot proceed with the republication of these images without clear consent from the original Flickr source.

Before we can proceed with your manuscript, we ask that you please address the following:

We require specific consent from the original copyright holder(s) to publish these images in PLOS ONE, under the CC BY 4.0 license.

To seek permission from the copyright owner(s) to publish these figures under the Creative Commons Attribution License (CCAL), CC BY 4.0, please contact them with the following text and PLOS ONE Request for Permission form ( http://journals.plos.org/plosone/s/file?id=7c09/content-permission-form.pdf):

“I request permission for the open-access journal PLOS ONE to publish XXX under the Creative Commons Attribution License (CCAL) CC BY 4.0 (http://creativecommons.org/licenses/by/4.0/). Please be aware that this license allows unrestricted use and distribution, even commercially, by third parties. Please reply and provide explicit written permission to publish XXX under a CC BY license.”

Please upload the granted permission to the manuscript as an "Other" file in Editorial Manager.

In the figure caption of the copyrighted figure, please include the following text: “Republished from [ref] under a CC BY license, with permission from [name of publisher], original copyright [original copyright year].”

Please note that RightsLink permission forms often impose use restrictions that are incompatible with our CC BY 4.0 license, and we are therefore unable to accept these permissions. For this reason, we strongly recommend contacting copyright holders with the PLOS ONE Request for Permission form.

If you are unable to obtain permission from the original copyright holder(s), or show that the original source clearly permits CC BY 4.0 reuse, please either remove the figures from the COCO Consortium, or supply replacement figures that comply with the CC BY 4.0 license.

**A1:**

Thank you for your response. Sorry, although we have contacted the Flick team, we are unable to obtain the copyright of the image under the CCBY4.0 license (only CCBY2.0 is published). To expedite the review process, we have decided to remove all original dataset images involving copyright issues from the paper. At present, all the images in our manuscript are schematics of our method, or theoretical visualizations of experimental results obtained through our method. There are no more copyright issues involved.

In addition, in the experimental part of our manuscript, there is a confusion matrix graph, which is the explanation of the failure cases that reviewer #1 asked us to include in the paper. Previously, we used raw images from the COCO2017 dataset for presentation in our paper. In consideration of image copyright issues, we have deleted these images in this revision and used this confusion matrix theory diagram to explain.

**Q2.** We note that your manuscript is not formatted using one of PLOS ONE’s accepted file types. Please reattach your manuscript as one of the following file types: .doc, .docx, .rtf, or .tex (accompanied by a .pdf).

If your submission was prepared in LaTex, please submit your manuscript file in PDF format and attach your .tex file as “other.”

**A2:** We have checked and modified our manuscript according to journal style requirements. Our manuscript is written in LaTex, so the manuscript is submitted in PDF format, and the corresponding LaTex files are uploaded in the form of compressed packages through the attachment.

**Q3.** Please note that your Data Availability Statement is currently missing the repository name. If your manuscript is accepted for publication, you will be asked to provide these details on a very short timeline. We therefore suggest that you provide this information now, though we will not hold up the peer review process if you are unable.

**A3：**Thank you for your feedback. The datasets used in this study are all publicly available. The COCO dataset can be accessed at https://cocodataset.org, and both the RTTS and SOTS datasets, which are part of the RESIDE public dataset, can be accessed at https://sites.google.com/view/reside-dehaze-datasets/. The links to the above public datasets have been added to the corresponding parts of our manuscript. The COCO dataset and RTTS dataset are suitable for object detection tasks, while the SOTS dataset is suitable for image dehazing tasks. Since these datasets are already hosted on stable, public repositories and are widely recognized in the research community, additional deposition in another repository is not necessary.

In addition, in order to promote academic exchange and ensure the repeatability of the research, we sorted out the procedures of the training and testing phase of the method proposed in our manuscript, and uploaded the procedures of the testing phase, the trained model weight file and the relevant experimental setup file to the GitHub repository. The repository will include comprehensive installation guidelines, usage instructions, and necessary dependency information to ensure that researchers can easily implement and replicate the methods presented in this article. We look forward to working with the broader research community to advance the application and development of object detection technologies in low-visibility environments.

**Q4.** "Thank you for uploading your study's underlying data set. Unfortunately, the repository you have noted in your Data Availability statement does not qualify as an acceptable data repository according to PLOS's standards.

At this time, please upload the minimal data set necessary to replicate your study's findings to a stable, public repository (such as figshare or Dryad) and provide us with the relevant URLs, DOIs, or accession numbers that may be used to access these data. For a list of recommended repositories and additional information on PLOS standards for data deposition, please see https://journals.plos.org/plosone/s/recommended-repositories. "

**A4:** Thank you for your feedback. The datasets used in this study are all publicly available. The COCO dataset can be accessed at https://cocodataset.org, and both the RTTS and SOTS datasets, which are part of the RESIDE public dataset, can be accessed at https://sites.google.com/view/reside-dehaze-datasets/. The links to the above public datasets have been added to the corresponding parts of our manuscript. The COCO dataset and RTTS dataset are suitable for object detection tasks, while the SOTS dataset is suitable for image dehazing tasks. Since these datasets are already hosted on stable, public repositories and are widely recognized in the research community, additional deposition in another repository is not necessary.

**Q5.** Please ensure that you refer to Table 3 in your text as, if accepted, production will need this reference to link the reader to the Table.

**A5:** Thank you for your feedback. Table 3 has already been referenced in the manuscript. Please let us know if further adjustments are needed.

**Q6:** Please note that PLOS utilizes the CC BY 4.0 license (https://creativecommons.org/licenses/by/4.0/) which means that all material on our website is freely available online, and any third party is permitted to access, download, copy, distribute, and use these materials in any way, even commercially, with proper attribution.

If the figures in your submission have already been published, authors must provide proper attribution, referencing the source clearly, and obtain permissions if the images are copyrighted.

Before we can proceed with your manuscript, we ask that you please clarify the following:

1) Please explain where the authors obtained the images in Figures 1, 6, 7, 8, & 9 in your submission or if the authors created the images themselves.

2) Please state whether Figures 1, 6, 7, 8, & 9 have been previously copyrighted.

3) If any of the images in Figures 1, 6, 7, 8, & 9 have been previously copyrighted, we require specific consent from the copyright holder(s) to publish these images in PLOS ONE, under the CC BY 4.0 license.

**A6:** Thank you for your inquiry. To ensure our article meets the journal's requirements and proceeds smoothly through the re-review process, we have removed the images originally included in Figures 6, 7, and 8, and made modifications to Figure 1. The image below shows the revised Figure 1.

**Q7.** Please ensure that your manuscript meets PLOS ONE's style requirements, including those for file naming. The PLOS ONE style templates can be found at

https://journals.plos.org/plosone/s/file?id=wjVg/PLOSOne_formatting_sample_main_body.pdf and https://journals.plos.org/plosone/s/file?id=ba62/PLOSOne_formatting_sample_title_authors_affiliations.pdf

**A7:** We have checked and modified our manuscript according to journal style requirements. Our manuscript is written in LaTex, so the manuscript is submitted in PDF format, and the corresponding LaTex files are uploaded in the form of compressed packages through the attachment.

**Q8.** Please note that PLOS ONE has specific guidelines on code sharing for submissions in which author-generated code underpins the findings in the manuscript. In these cases, we expect all author-generated code to be made available without restrictions upon publication of the work. Please review our guidelines at

https://journals.plos.org/plosone/s/materials-and-software-sharing#loc-sharing-code and ensure that your code is shared in a way that follows best practice and facilitates reproducibility and reuse.

**A8：**In order to promote academic exchange and ensure the repeatability of the research, we sorted out the procedures of the training and testing phase of the method proposed in our manuscript, and uploaded the procedures of the testing phase, the trained model weight file and the relevant experimental setup file to the GitHub repository. The repository will include comprehensive installation guidelines, usage instructions, and necessary dependency information to ensure that researchers can easily implement and replicate the methods presented in this article. We look forward to working with the broader research community to advance the application and development of object detection technologies in low-visibility environments.

**Q9.** We note that the grant information you provided in the ‘Funding Information’ and ‘Financial Disclosure’ sections do not match. When you resubmit, please ensure that you provide the correct grant numbers for the awards you received for your study in the ‘Funding Information’ section.

**A9:** We have verified and revised the "Funding Information" section as follows:

This work was supported by the Natural Science Foundation of Shandong Province under Grant ZR2021MF031.

**Q10.** Thank you for stating the following financial disclosure:  "the Shandong Provincial Natural Science Foundation of China" Please state what role the funders took in the study.  If the funders had no role, please state: ""The funders had no role in study design, data collection and analysis, decision to publish, or preparation of the manuscript.""  If this statement is not correct you must amend it as needed.  Please include this amended Role of Funder statement in your cover letter; we will change the online submission form on your behalf.

**A10:** We have added the following in the cover letter:

**Funding:** This work was supported by the Natural Science Foundation of Shandong Province under Grant ZR2021MF031. The funders had no role in study design, data collection and analysis, decision to publish, or preparation of the manuscript.

**Competing interests:** The authors have declared that no competing interests exist.

**Q11:** We note that Figures 1, 6 and 7 in your submission contain copyrighted images. All PLOS content is published under the Creative Commons Attribution License (CC BY 4.0), which means that the manuscript, images, and Supporting Information files will be freely available online, and any third party is permitted to access, download, copy, distribute, and use these materials in any way, even commercially, with proper attribution. For more information, see our copyright guidelines: http://journals.plos.org/plosone/s/licenses-and-copyright.
 We require you to either (1) present written permission from the copyright holder to publish these figures specifically under the CC BY 4.0 license, or (2) remove the figures from your submission:
 a. You may seek permission from the original copyright holder of Figures 1, 6 and 7 to publish the content specifically under the CC BY 4.0 license. 
 We recommend that you contact the original copyright holder with the Content Permission Form (http://journals.plos.org/plosone/s/file?id=7c09/content-permission-form.pdf) and the following text: “I request permission for the open-access journal PLOS ONE to publish XXX under the Creative Commons Attribution License (CCAL) CC BY 4.0 (http://creativecommons.org/licenses/by/4.0/). Please be aware that this license allows unrestricted use and distribution, even commercially, by third parties. Please reply and provide explicit written permission to publish XXX under a CC BY license and complete the attached form.”
 Please upload the completed Content Permission Form or other proof of granted permissions as an ""Other"" file with your submission. 
In the figure caption of the copyrighted figure, please include the following text: “Reprinted from [ref] under a CC BY license, with permission from [name of publisher], original copyright [original copyright year].”
 b. If you are unable to obtain permission from the original copyright holder to publish these figures under the CC BY 4.0 license or if the copyright holder’s requirements are incompatible with the CC BY 4.0 license, please either i) remove the figure or ii) supply a replacement figure that complies with the CC BY 4.0 license. Please check copyright information on all replacement figures and update the figure caption with source information. If applicable, please specify in the figure caption text when a figure is similar but not identical to the original image and is therefore for illustrative purposes only.

**A11:** Thank you for your inquiry. To ensure our article meets the journal's requirements and proceeds smoothly through the re-review process, we have removed the images originally included in Figures 6, 7, and 8, and made modifications to Figure 1. The image below shows the revised Figure 1.

**Review Comments to the Author**

**Reviewer #1**

**Q1.** Authors compare the proposed technique with existing techniques in terms of detection accuracy. Authors should also compare the proposed technique with existing techniques in terms of computation times, training times and storage sizes.

**A1:** *Thanks for the reviewer's comments. We have added a comparison of existing techniques with other methods in terms of detection accuracy, computation time, training time, and storage size. Please see our revised manuscript. All revisions have been in blue.*

*Page 17, the section of Experimental Results:*

**Q2.** The failure case of the proposed technique must be described and discussed in the text.
**A2:** *Thanks for the reviewer's comments. We have added the discussion on failure cases of proposed methods. Please see our revised manuscript. All revisions have been in blue.*

*Page 16 and 17, the section of Experimental Results:*

**Q3.** The source code of the proposed technique must be shared to implement it easily.
**A3:** *Thanks for the reviewer's comments. To foster academic exchange and ensure research reproducibility, we plan to upload the source code and relevant experimental setup files of this paper to a GitHub repository upon its acceptance. This repository will include comprehensive installation guides, usage instructions, and necessary dependency information, ensuring that researchers can easily implement and reproduce the proposed method. We look forward to collaborating with the broader research community to advance the application and development of object detection techniques in low-visibility environments. Please see our revised manuscript. All revisions have been in blue.*

*Page 1, the section of Abstract:*

**Q4.** Authors should cite the following recent object detection paper [Azadvatan2024arXiv].
**A4:** *Thank you for your suggestion. We will carefully consider the findings of Azadvatan2024arXiv and include the relevant citation in our paper to ensure that our work fully reflects the latest developments in this field. All revisions have been in blue.*

*Page 6, the section of Introduction:*

**Q5.** At the end of Conclusion section, authors can also give some important future directions to the readers, researchers.

**A5:** *Thanks for the reviewer's comments. We have added some discussions about future directions to the readers, researchers. All revisions have been in blue.*

*Page 21 and 22, the section of Conclusion:*

**Reviewer #2**

**Q1:** There is no comparison between the proposed method and other state-of-the-art methods specifically designed for haze conditions. The authors should include a more detailed discussion and comparative analysis, explaining why the proposed method outperforms others.

**A1:** *Thanks for the reviewer's comments. We agree that the lack of a comparison with existing state-of-the-art methods specifically designed for haze conditions is a shortcoming. In the revised version, we will include more comparative experiments with the latest dehazing methods, and we will conduct the experiments using the RESIDE public dataset.All revisions have been in blue.*

*Page 17 and 19, the section of Experimental Results:*

**Q2:** I suggest covering more benchmark metrics for comparison.

**A2:** *Thanks for the reviewer's comments. In the revised version, we have reintroduced the mAP50 and mAP75 metrics for the object detection task, and additionally included the PSNR and SSIM metrics for the image dehazing task. All revisions have been in blue.*

*Pages 15 , 16 and 19, the section of Quantitative Evaluation:*

**Q3:** The conclusion of this paper lacks future directions or suggestions, which are necessary for guiding further research.

**A3:** *Thanks for the reviewer's comments. We have added some discussions about future directions to the readers, researchers. All revisions have been in blue.*

*Page 21 and 22, the section of Conclusion:*

**Q4:** The manuscript could be strengthened by including experiments on additional datasets, particularly those that capture real-world haze conditions. Alternatively, the authors could discuss the potential limitations of the current approach when applied to real-world data and suggest future research directions to address these challenges.

**A4:** *Thanks for the reviewer's comments. To further enhance the completeness of this paper, we have added experiments on the RTTS real-world haze image dataset. All revisions have been in blue.*

*Page 16, the section of Experimental Results:*

**Reviewer #3**

**Q1:** In the introduction and related work sections, some of the relevant works are missing, especially the works about the evaluation of haze and dehazing, for example, HazDesNet: an end-to-end network for haze density prediction; Quality evaluation of image dehazing methods using synthetic hazy images; Objective quality evaluation of dehazed images.

**A1:** *Thanks for the reviewer's comments. Please see our revised manuscript. We recognize the need for more in-depth analysis and discussion in the Related Work of the paper. We have carefully reviewed these articles and cited them in relevant sections. All revisions have been in blue.*

*Page 4, the section of Deep learning-based image dehazing technology:*

**Q2:** “…This degradation in image quality can severely impact the accuracy of computer vision tasks, such as object detection…”
“…Eliminating haze from images not only improves image quality but also enhances information readability…”
Some related surveys are suggested to be given for better referring of the related topics, for example visual quality as its evaluation as discussed in the related surveys, e.g., ‘Perceptual image quality assessment: a survey’, ‘Perceptual video quality assessment: a survey’, and ‘Screen content quality assessment: Overview, benchmark, and beyond’.

**A2:** *Thanks for the reviewer's comments. We have enriched the content of the literature review in the revised manuscript. All revisions have been in blue.*

*Page 5, the section of Deep learning-based image dehazing technology:*

**Q3:** The experimental validation needs to be strengthened. For example, the combinations of SOTA dehazing methods and SOTA detection methods are also suggested to be included into the comparison.

**A3:** *Thanks for the reviewer's comments. In the revised version, we have added experiments comparing our method with the combination of state-of-the-art dehazing methods and state-of-the-art detection methods. All revisions have been in blue.*

*Page 18 and 19, the section of Ablation experiment:*

**Q4:** The formats of some references are suggested to be double-checked.

**A4:** Thanks for the reviewer's comments. We have carefully checked the format of all references and made revisions.

**Reviewer #4**

**Q1:** The experimental design is not detailed enough to substantiate the theoretical claims, with inadequate information in the code and experiment sections.

**A1:** *Thanks for the reviewer's comments. To complement the theoretical claims of our experiment, we added target detection experiments in real-world foggy conditions using the RTTS public dataset. Additionally, we conducted control experiments for image dehazing using the RESIDE public dataset. Finally, we introduced new evaluation metrics, including mAP, mAP75, PSNR, and SSIM, to enrich our experimental analysis. We also compared our approach with the latest methods that combine dehazing models and object detection models. We plan to upload the source code and relevant experimental setup files of this paper to a GitHub repository upon its acceptance. All revisions have been in blue.*

*Page 1, 16, 18 and 19, the section of Experimental Results:*

**Q2:** The paper lacks a focus on parameter fine-tuning, which is critical for evaluating the model's robustness.

**A2:** *Thanks for the reviewer's comments. We conducted additional ablation experiments to fine-tune the parameters of the Wise-IoU method. All revisions have been in blue.*

*Page 20 and 21, the section of Ablation experiment:*

**Q3:** There is no discussion of the computational costs, especially during inference, which is an important aspect to consider.

**A3:** *Thanks for the reviewer's comments. We have added a comparison of existing techniques with other methods in terms of detection accuracy, computation time, training time, and storage size. Please see our revised manuscript. All revisions have been in blue.*

*Page 17 and 18, the section of Experimental Results:*

**Q4:** The literature review is not comprehensive enough, failing to provide sufficient comparisons with established methods.

**A4:** *Thanks for the reviewer's comments.We have reorganized the relevant methods and made additional improvements. All revisions have been in blue.*

*Page 4, 5 and 6, the section of Related work:*

**Q5:** The absence of standard deviations in the reported results undermines their statistical validity.

**A5:** *Thanks for the reviewer's comments. To address the omission of standard deviation in the reported results, we calculated the standard deviation for our model's outcomes. All revisions have been in blue.*

*Page 16, the section of Experimental Results*

**Q6:** The manuscript does not address how the model generalizes to unseen data, which is crucial for its practical use.

**A6:** *Thanks for the reviewer's comments. We have added future work on unseen data in our outlook. All revisions have been in blue.*

*Page 21 and 22, the section of Conclusion:*

**Q7:** There is a lack of discussion on potential limitations and areas for future research, leaving the reader with an incomplete picture of the work's scope.

**A7:** *Thanks for the reviewer's comments. We have added some discussions about potential limitations and future directions to the researchers. All revisions have been in blue.*

*Page 21 and 22, the section of Conclusion:*

**Q8:** What exactly sets your novel method apart? Is it akin to a hypothetical 'YOLOv9'? The paper claims to enhance object detection in low-visibility haze conditions with YOLOv9s, but it is essential to validate this claim by using more public datasets. Proving that the proposed model is truly the best requires thorough comparative experiments.

**A8:** *Thanks for the reviewer's comments. To further enhance the completeness of this paper, we have added experiments on the RTTS real-world haze image dataset. All revisions have been in blue.*

*Page 16, the section of Experimental Results:*
