# Supplementary material for: Enhanced object detection in low-visibility haze conditions with YOLOv9s
Source: PLoS One. 2025 Feb 24;20(2):e0317852. doi: 10.1371/journal.pone.0317852 (PMC11849987; doi:10.1371/journal.pone.0317852)
Supplement: Attachment — Submitted filename: response_to_reviewers_auresp_2.docx [file pone.0317852.s004.docx]

**Original Manuscript ID:** PONE-D-24-31889R2

**Original Article Title: “**Enhanced Object Detection in Low-Visibility Haze Conditions with YOLOv9s”

**To:** PLOS ONE Editor

**Re:** Response to reviewers

Dear Editor,

Thank you for allowing a resubmission of our manuscript, with an opportunity to address the reviewers’ comments.

We are uploading (a) our point-by-point response to the comments (below) (Response to Reviewers). However, (b) an updated manuscript with blue highlighting indicating changes (Revised Manuscript with Track Changes) and (c) an updated manuscript without blue highlighting indicating changes (Manuscript) have not changed, so the content of the two files uploaded is the same, only the file name is different.

Best regards,

Bin Zhou et al.

**Review Comments to the Author**

**Reviewer #1**

**Q1.**In this paper, authors introduce an innovative object detection framework designed to enhance visibility and accuracy in low-visibility haze conditions. The approach begins by applying haze to the COCO dataset and then employs a dehazing module that leverages an improved GAN with an integrated patchwise contrastive loss function to generate dehazed images. These images are subsequently fed into an advanced YOLOv9s object detection model, which has been refined with the addition of the Efficient Multi-Scale Attention module and the Wise-IoU loss function to boost detection precision. Comprehensive experiments conducted on the hazed COCO dataset demonstrate the superiority of the proposed model in terms of detection accuracy. In the revised paper, it seems that authors handled most of the reviewer comments into account. However, there are some minor issues, which need to be handled in the text properly:

Authors compare the proposed technique with existing techniques numerically. Authors should also compare the proposed technique with existing techniques visually.

**A1:**

Thank you for your feedback. We will provide a thorough response below.

We have included visualizations in our response to reviewers that compare our method with others on the COCO dataset test set. These visualizations intuitively showcase the detection results of our method versus others. We randomly select five test images from the COCO dataset to create five sets of visualization results, which are displayed below in detail. For example, in the first set, our method achieves better accuracy compared to other methods and does not experience missed detections as seen in methods such as YOLOv8s and YOLOv5s. Additionally, compared to the original labels, our method does not make any incorrect predictions. From these visualizations, it is evident that our method achieves better overall detection accuracy and has lower probabilities of missed and false detections compared to other methods. However, we don’t include these images in our paper; instead, they are available on our open-source GitHub repository at the link (https://github.com/PaTinLei/EOD) for readers’ reference and study.

Next, we will explain why we don’t include these visualizations in the paper.

Firstly, in the previous revision responses, we were required to provide CC BY 4.0 license declarations for all copyrighted images. We made various efforts, including but not limited to researching materials, contacting dataset officials, providing CC BY 2.0 licenses, and replacing datasets. However, we were ultimately unable to obtain the CC BY 4.0 license for the datasets involved in our method. This is difficult because these public datasets are released without declaring a CC BY 4.0 license, and their team only offers a CC BY 2.0 license. To facilitate our paper's swift entry into the review process, we have to remove all images involving copyright issues from the manuscript.

Secondly, we also considered creating our own dataset. However, producing a dataset ourselves would require a significant amount of time for data collection and annotation, which is overly burdensome for an individual. Furthermore, our own dataset would not be as reference-worthy or reliable as public datasets. Moreover, we believe that even our own dataset would still present copyright issues, such as copyrighted elements within the images.

Finally, considering the reasons mentioned above, we don’t include images with copyright disputes in our paper. We hope that by providing this response, we can ensure a clear presentation of the visual results from our manuscript.
